# Masculinity and immune system efficacy in men

**DOI:** 10.1371/journal.pone.0243777

**Published:** 2020-12-14

**Authors:** Judyta Nowak-Kornicka, Barbara Borkowska, Bogusław Pawłowski

**Affiliations:** Department of Human Biology, University of Wrocław, Wrocław, Poland; Ehime University Graduate School of Medicine, JAPAN

## Abstract

Masculinity-related morphological traits are supposed to be honest indicators of a man's biological quality. While some studies showed that sexually dimorphic traits are related to various aspects of biological condition such as general health, immunity or fertility, still little is known about the relationship between masculine traits and the effectiveness of innate and adaptive immunity in humans. The aim of this study was to see if masculine traits, which are dependent on androgen levels in foetal and pubertal stages of development, are related to the immune quality in healthy men. The immune quality was evaluated for 91 healthy men aged 19–36 years. Immunity measurements included innate and adaptive parameters. General health status, age, testosterone level, BMI, physical activity, and smoking were controlled. The shoulder-to-hip ratio (SHR), 2D:4D digit ratio and hand-grip strength (HGS) were used as markers of masculinization. The regressions showed that when controlling for confounds, masculinity-related traits were in general not related to innate and adaptive immunity. Only a weak association was observed for right 2D:4D ratio and T-lymphocyte counts (but it becomes non-significant after adjustment for multiple comparisons). Our results do not support the premise that masculinity is a cue for immunological quality in men. However, the positive association between right 2D:4D and T lymphocytes might suggest that further studies are needed to verify if androgen stimulation in prenatal development might be related to immunity in adulthood.

## Introduction

Darwin's (1871) [1] theory of sexual selection posits that sexually dimorphic traits are adaptive as they are involved in intrasexual competition and intersexual choice. Individuals with highly expressed sexually selected traits can more effectively attract a member of the opposite sex and therefore reach higher reproductive success, having more and healthier offspring (especially men). Some evolutionary hypotheses, such as the good genes hypothesis, suggest that sexually dimorphic traits can be linked with various aspects of an individual's biological condition including immune system effectiveness [2, 3]. Traits such as masculinity in men or femininity in women, are sex-typical characteristics dependent on sex hormone proportions and are developed prenatally or mostly at puberty. In men, a higher testosterone level is usually related to higher masculinization (higher expression of masculinity/masculine traits). Having effective body defence mechanisms means having low susceptibility to infections, which is crucial for survival, and is therefore a very important determinant of fitness. Thus, if in accordance to the hypothesis that physically attractive (dimorphic) traits are the cues of biological condition, such traits (at least theoretically) should be linked with immune functioning. This would also mean that women’s preferences for highly masculine males may in consequence lead to selection for such male traits that signal viability benefits (e.g. immune quality) for offspring. Other hypotheses considering the possible mechanisms to explain of the relationship between sexually dimorphic expression and biological quality, indicate that these traits might be costly to develop and maintain [4]. It is also worth noting that intrasexual male-male competition is also usually based on such characters (e.g. body size and strength) that are costly to produce and therefore are supposed to signal biological quality. The evolution of traits that are fundamental for male intrasexual competition (e.g. fighting ability) or related to hunting ability [5] and lead to success in mating competition should be favoured by natural and sexual selection [6]. The question is whether, in accordance to the handicap hypothesis [4], such traits are also related to the physiological cost borne by an organism. Folstad and Karter (1992) [7] were the first to suggest that testosterone i.e. the hormone influencing development of masculine traits, negatively affects immunity. They called it the immunocompetence handicap hypothesis (ICHH). In support of this, various studies that have tried to verify the proposed proximate mechanisms linking masculinization and immune system functioning have shown that testosterone might in fact have immunosuppressive [8–10] and prooxidant properties [11, 12].

According to the ICHH assumption, only individuals with a high quality immune system can produce and maintain high levels of immunosuppressive testosterone and develop a high degree of masculinity without a reduction in fitness. To date, however, the results of the studies on ICHH assumptions are mixed and not at all conclusive, suggesting that testosterone-immune interactions are still in question [9, 13–22]. Recent studies testing innate and adaptive immune parameters showed, for instance, that blood-circulating androgen concentration was in general not associated with the effectiveness of an individual's innate and adaptive immune function. Furthermore, the most potent androgens (free testosterone) appeared to be positively associated with the strength of a post-vaccination response [21]. However, the authors point out that circulating androgen levels are strongly influenced by lifestyle-associated factors (such as diet, stress and sporting activity [23, 24], fatherhood [25] or paternal care [26] and therefore might not reflect general immune quality. It is also possible that immune functioning is affected by androgens in early life development, mainly in the prenatal period when they exert an organizational effect on immune cells [27] or during puberty when they influence both masculinization and immunity [28–30]. It is likely that, masculine traits, commonly used as proxy markers of prenatal or pubertal testosterone exposure, might be better indicators of biological quality than lifestyle dependent hormone concentrations measured in adulthood. However, some studies have shown that men whose faces were rated as more masculine produced higher levels of specific antibodies in response to the hepatitis B vaccine [15] and had a stronger cytokine response [19]. Furthermore, men with deeper, more monotone voices had higher levels of secretory immunoglobuline A [20]. Despite the general assumption that masculine traits are linked with immune quality, several papers (including meta-analyses) yield conflicting results, showing either very weak or no evidence supporting ICHH [13, 16–18]. Therefore, the question of whether masculinization can be commonly used as a marker of biological quality, especially when considering immunity, is then still unanswered. In addition, there is also little known about the relationship between various immune parameters and bodily traits of masculinity developed under the influence of androgens during foetal development or during puberty.

In this study we used three markers of masculinization that are commonly employed as testosterone-dependent markers of biological condition i.e. 2D:4D digit ratio (2D:4D), the shoulder-to-hip ratio (SHR) and hand-grip strength (HGS). The **2D:4D digit ratio** (2^nd^ digit length in relation to 4^th^ digit length) is supposed to be a proxy biomarker of prenatal androgen exposure and in many studies is used as a marker of masculinization [31], though this is also not without criticism. However, Richards et al. (2019) [32] did not find a relationship between 2D:4D ratio and prenatal testosterone (PT) in male infants and Richards (2017) [33] further describes controversies on the research methodology related to 2D:4D ratio and PT level. However, it is known that in general men have a lower-digit 2D:4D ratio than women [34], and additionally, digit ratio has been found to be related to, for instance, semen quality [35], facial masculinity [36], and perception of a man's masculinity and dominance [37]. Its relation to circulating testosterone levels in adults, however, is questioned [35, 37, 38].

**SHR** (shoulder width divided by hip width) is one of the most widely used body markers of androgen exposure during puberty, when skeletal development is strongly affected by sex hormones. In males relatively greater shoulder width in relation to the hips is a result of androgen stimulation on cartilage cells in the shoulder [39]. Consequently, men's body shape is characterized by broader shoulders and narrower hips. It has been shown that in males SHR is correlated with such masculine traits as hand-grip strength [40] and sexual behaviour (e.g. age at first sexual intercourse or number of sex partners [41, 42].

**HGS** (hand-grip strength) is a marker of masculinization reflecting overall body strength [43]. HGS in males significantly increases at puberty and becomes highly sexually dimorphic [44, 45]. The largest increase in HGS is observed between 10–20 years old, with this remaining stable for up to 40–50 years [46]. This suggests that the change in T level occurring at puberty is one of the main factors that determines HGS in men. The connection between actual circulating testosterone concentration and HGS has been verified in various groups. For example, experimental studies conducted on older or hypogonadal men show that reduced HGS is associated with lower testosterone levels [47–49], and that androgen supplementation increases HGS [48]. However, the results of several correlational studies are inconclusive, with findings showing that HGS has been found to be positively correlated [50, 51], but also unrelated to testosterone levels [52, 53]. Other studies have also shown that HGS is associated with masculinity related morphological and behavioural traits, such as shoulder breadth [40], 2D:4D digit ratio [54, 55], age at first sexual intercourse and number of sexual partners [40], measures of aggression and social competition [40, 56], and perceptions of a man's facial masculinity and dominance by women [57]. These observations might suggest that, in general, HGS is mostly related to pubertal (or around pubertal) androgens levels [46], but that it might also be sensitive to testosterone variations outside of puberty, as has been shown in various correlational and experimental studies.

Investment in masculine traits is an important component of male reproductive effort including inter and intra-sexual selection. Thus, the observation that masculine traits (as a component of physical attractiveness) might be related to immune quality could provide evidence that sexual selection based on testosterone-dependent traits has important biological implications. The relationship between testosterone-dependent traits and immune system functioning has already been tested many times in non-human animals (mostly in birds) showing inconsistent results [58–61]. Only a few correlational studies in humans have tried to verify these associations using selective masculine traits (mostly facial ones) and usually only indirectly measuring immunity (self-reported frequency of infections see [16, 62]) or with a limited selection of immune parameters [15, 19, 63]. To the best of our knowledge, apart from the study on body height [64], there are no studies on the direct relationship between body masculinity and various immune functions whilst controlling for testosterone concentration, age or BMI in men.

The aim of our study was to test if a man's masculinization can be an indicator of immune quality. To evaluate immune system functioning we used a number of cell-mediated and humoral-mediated innate and adaptive immune parameters, constituting various immune mechanisms. We hypothesize that if testosterone dependent masculinity is a cue of biological condition, only individuals with well functioning immunity should be characterized by higher masculinity values of such markers as 2D:4D, SHR and HGS.

## Materials and methods

### Ethics statement

The research was approved by the Bioethics Commission at the Lower Silesian Chamber of Physicians and Dentists’ ethics committee (2/PB/2013). All participants read and signed the informed consent form. All medical procedure was consistent with the guideline included in the “Declaration of Helsinki–Ethical Principles for Medical Research Involving Human Subjects” formulated by World Medical Association in 2013 (https://www.wma.net/policies-post/wma-declaration-of-helsinki-ethicalprinciples-for-medical-researchinvolving-human-subjects/).

### Participants, selection criteria and study procedure

The participants were one hundred and thirty-four healthy men, aged 19–36. They were recruited to the project by local newspapers and radio advertisements. The participation criteria were: no chronic diseases, no current health problems, no hormonal disorders or supplementation, and consent to vaccination against seasonal influenza. Among recruited men, forty-three were excluded from the final study group according to the following criteria: had elevated inflammatory markers (C-reactive protein levels > 5mg/mL), liver enzymes (both >40U/L) or glucose levels (>120mg/dL) (N = 7), had outstanding values of BMI (N = 3), had incomplete immunological, morphological or questionnaire data (N = 28), declared infection during the study (N = 3), or had not completed the full study procedure (N = 2). The final analyses, including immune factors, were performed for 91 healthy men aged 19–36 (mean age 27.02, SD+/- 4.80). Phagocytic-uptake analyses and lymphocyte-proliferation response were performed for smaller sample sizes (N = 81 and N = 54 respectively). The majority of participants lived in Wroclaw (PL) or in smaller cities (73%), had university education level or were students (90%). The study group was also quite homogeneous with regard to socioeconomic status (SES), and the majority (99%) declared that their current financial status was at least average.

The study group were subjected to the following procedure: a) during the first visit: medical examination for general health-status evaluation and qualification for vaccination, blood collection for basic biochemical analyses, hormonal and immunological analyses, vaccination against flu, anthropometric measurements and some questionnaires; b) during the second visit (which was performed about 28 days after the vaccination) only a blood sample was collected to evaluate the strength of the immune response to the vaccine, and some questionnaires were filled in. Both the medical procedure (medical examination, vaccination and blood collection) and anthropometry were performed in a private medical clinic in Wroclaw, Poland. Blood samples were collected from the participants between 7:30–9:00 a.m., gently mixed and remained at room temperature until processing. Within two hours from the collection, the samples were transported (in a blood-transport container) to the Institute of Genetics and Microbiology for further immunological and hormonal analyses, whereas biochemical tests of blood were commissioned to be performed in a certified analytical laboratory (DIAGNOSTYKA®). In the laboratory, fresh blood samples were immediately used for different tests (phagocytic uptake, ROS production, lymphocyte count and lymphocyte-proliferation response). Blood samples collected in serum vacutainers were centrifuged, then the separated serum was portioned into sterile eppendorf tubes and frozen at -80°C. Serum samples for hormones, complement activity, and lysozyme-activity determination were stored for max. 3 months from collection, whereas serum samples for antibody-level determination (IgA, IgG, anti-flu) were stored for max. 6 months from collection. Due to the fact that all samples were collected at the same early morning time (between 7:30–9:00 a.m.), we assumed that hormone-level fluctuations are negligible (non-significant) and therefore we did not register the precise time (hour) when the blood samples were collected.

During the process of participants’ selection for final analyses, it was necessary to exclude those with any health problems including elevated markers of inflammation that might signal asymptomatic infection or those who declared any infection symptoms. Consequently, a higher level of any immune parameter might be misinterpreted as better immune quality rather than ongoing immune activation in response to an infection. In addition, we had to exclude participants who declared an infection and who reported using antibiotics between the two visits as it might have affected the post-vaccination response.

The participants' recruitment and vaccination were performed in two consecutive flu seasons: from September to March in 2013/2014 and 2014/2015. Participants received the seasonal influenza vaccine (Vaxigrip, SanofiPasteur®), containing the virus particle recommended by the World Health Organization for each season.

Participants of this study were a group of men recruited also for the project on attractiveness, sex hormones and immunity measures in humans [see 21].

### Body and strength measurements

#### The second to fourth digit ratio (2D:4D)

Finger lengths were directly measured using digital calipers accurate to 0.01 mm, twice on both hands. The 2D:4D ratio was calculated separately for each hand.

#### Shoulder to hip ratio (SHR)

Shoulder width was measured using anthropometric spreading calipers as biacromial width (*acromiale-acromiale*), whereas hip width was measured as bi-iliocristal diameter (*iliocristale-iliocristale*). All measurements were taken three times and the mean figures were used to calculate both digit ratio and SHR.

#### Hand-grip strength (HGS)

Measured by a hand dynamometer (Baseline®). The participants were instructed to squeeze the dynamometer as tightly as possible. Two separate squeezes from each hand were collected, and then the maximum values (measured in kilograms) from all measurements were used in the statistical analyses.

#### Body mass and height

Double measured and averaged.

### Immunological analyses

#### Innate immune parameters

*Complement system*. The group of proteins present in human body fluids which when activated leads to cell lysis. The total complement activity in a zymosan-activated participant's serum was measured using commercial kits (MicroVue, QUIDEL) and spectrophotometers: Asys UVM 340 (Biochrom®).

*Lysozyme*. The most abundant enzyme present in human serum with strong antibacterial properties. The serum antibacterial activity to lysis *Micrococcus lysodeicticus* was measured using a turbidimetric assay. The absorbance were measured on spectrophotometers: Asys UVM 340 (Biochrom®).

*Neutrophils*. The group of professional phagocytes constituting the first line of immune defence. Neutrophils' ability to absorb/engulf pathogens (phagocytic uptake) and eliminate them by reactive oxygen-species formation was measured using commercial kits (PHAGOTEST, Glycotope®), and the chemiluminescence method respectively. The results were measured on an FASC Calibur (BD®) and calculated using BD CellQuest software.

#### Adaptive immune parameters

*Lymphocytes T and B*. The crucial adaptive effector cells responsible for antibody production (B cells) and memory-cell development. Total numbers of circulating T cells and B cells were measured using commercial kits (TriTest, BD®), measured on an FASC Calibur (BD®) and calculated using BD CellQuest software.

*Immunoglobulin A and G*. The major classes of antibodies present in human serum that are able to bind antigens with high specificity. Antigen-antibody complexes are then eliminated by multiple immune mechanisms. The total levels of immunoglobulins were measured using an enzyme-linked immunosorbent assay.

*The strength of post-vaccination response*. A gold standard to measure an organism's ability to produce specific antibodies in response to foreign antigens (a functional test of B-cell efficacy). The level of specific antibody level was measured using an enzyme-linked immunosorbent assay, the strength of post-vaccination was expressed as a fold increase from pre- to post-vaccination.

*Proliferation response after mitogen stimulation*. Reflects the ability of lymphocyte T to proliferate in response to a stimulus, which is a crucial function of cell-mediated adaptive immunity, used in clinical practice. The functional tests of T cells were performed using the most common stimulants–mitogens (concanavalin A and pokeweed) and a [3H] thymidyne-incorporation assay. Results were expressed as a stimulation index (SI).

All immune functions were evaluated by the methods precisely described in supplementary information in Nowak et al. (2018).

### Hormonal analyses

Free-testosterone levels were measured once, in serum obtained from blood samples collected in the morning hours (7:30–9:00 a.m.) and evaluated in a laboratory at the University of Wroclaw, by an enzyme-linked immunosorbent assay using appropriate commercial kits (DERMEDITEC®, cat no DE2924). The standards (included in each assay) and samples were assayed in duplicate in accordance to supplemental assay protocols. The intra- and inter-assay coefficient of variation were respectively: <10%, <10%, with the assay sensitivity 0.06 pg/ml. The absorbance was measured using a microplate reader, and spectrophotometers: Asys UVM 340 (Biochrom®) and λ = 450nm. The results were calculated in relation to the standard curve and expressed in pg/mL.

### Controlled factors

Body Mass Index (BMI) was calculated as weight [kg] divided by height [m] squared.

Smoking status and sports activity were self-reported. Participants were classified as non-smokers (N = 71) and smokers (N = 20) (regular or occasional). The sports group (N = 55) included men who declared practising sports in contrast to the non-practising-sports group (N = 36). There were no professional athletes in the whole study group.

Because of seasonal recruitments (some of the participants were recruited from September 2013 to March 2014 (months when flu vaccination is recommended in Poland), whereas others were recruited in the next "flu season" from September 2014 to March 2015), the majority of immunological analyses were performed using kits or reagents with different LOT numbers, which is why we also included the study season (I -2013/2014, or II -2014/2015) in the analyses.

### Statistical analyses

All continuous variables were log transformed for normalization of the residual distribution in regression models. The inter-correlations between immune functions or masculinity markers were tested using log-transformed data and Pearson's correlation. The relationship between immunity parameters and potential confounding variables (the participant's age, BMI, free-testosterone level, smoking status, sports activity and season of study), and masculinity markers and potential confounding variables (the participant's age, BMI, androgens, smoking status and sports activity) were tested using linear regression. The relationship between immune functions and HGS, SHR, 2D:4D digit ratio, participant's age, BMI, free testosterone, smoking status, sports activity and season of the study were tested using linear or multiple regression analysis controlling for confounding variables. The analyses were carried out using Statistica 12 (StatSoft Incorporated).

## Results and discussion

Descriptive statistics are presented in Table 1. The majority of immune function measures were not correlated or only weakly correlated with each other, except for the strong correlation between T and B-lymphocyte count (r = 0.73, p<0.01) and between Con A and PWM (r = 0.81; p<0.01) (see S1 Table in S1 File).

**Table 1:** Descriptive statistics for the analysed traits and all controlled variables.

		Mean	SD	min-max
age (year)		27.02	4.80	18.97–36.72
max HGS (kg)		51.98	8.45	30.00–83.00
SHR		1.51	0.12	1.27–1.77
right 2D:4D		0.98	0.03	0.90–1.08
left 2D:4D		0.98	0.03	0.93–1.05
BMI (kg/m^2^)		23.23	2.73	16.87–29.69
Body height (cm)		178.50	6.87	149.15–195.00
fT (pg/ml)		23.67	10.25	2.53–60.70
Innate immunity	complement activity (μg/ml)	185.85	57.77	47.14–287.53
	lysozyme activity^1^	0.4	0.09	0.08–0.6
	ROS production^2^	8.4	7.80	2.47–59.6
	phagocytic uptake^3^^+^	158.2	36.88	54.30–254.2
Adaptive immunity	T lymphocyte count (cells/μl) (cells/μl).	1474.23	555.68	338.05–3422.81
	B lymphocyte count (cells/μl) (cells/μl).	240.62	126.91	45.33–639.93
	IgA level (g/L)	1.97	1.08	0.59–7.34
	IgG level (g/L)	11.86	4.74	4.16–26.95
	strength of flu post-vaccination response^4^	7.87	9.75	1.00–64.00

1. The difference in absorbance value between control samples (bacteria suspension without lysozyme) and test samples (bacteria treated with serum-contained lysozymes).

2. Mean area under the chemiluminescence curve (AUC_CL_) for stimulated test sample divided by AUC_CL_ for control.

3. Mean fluorescence intensity of blood phagocytes after phagocytosis of fluorescently labelled bacteria.

4. Fold increase in antibody titers from pre to post-vaccination

5. Stimulation index − (CPM for stimulated test sample divided by CPM for unstimulated control).

+data for smaller sample size (N = 81 for phagocytic uptake. N = 54 for proliferation response to mitogen stimulation)

### The relationship between immune function and potential confounding variables

In the first step we tried to track potentially confounding variables that could affect measured immunity parameters. Using linear regression we analysed the relationship between each immune parameter and commonly known immunomodulatory factors such as the participant's age, BMI, free testosterone, smoking status, sports activity or study season. The factors that were related to any immune parameter were then controlled for in subsequent multiple regression models.

Among innate immune parameters only the neutrophil function (ROS generation) (β = 0.32; p = 0.002) was related to the study season. ROS generation was also predicted by sports activity (β = 0.21; p = 0.04). Among adaptive immune functions, both T and B-lymphocyte numbers were predicted by age (β = -0.30; p<0.01 and β = -0.24; p = 0.02 respectively) and the season (β = 0.35; p<0.001 for T cells and β = 0.34; p = 0.001 for B cells). IgG levels were related to the study season (β = 0.44; p<0.001) and BMI (β = -0.22; p = 0.03). The strength of post-vaccination response was predicted by age (β = -0.30; p = 0.004) and free-testosterone level (β = 0.24; p = 0.02).

Complement activity, lysozyme activity, phagocytic uptake, IgA levels and lymphocyte proliferation in response to ConA and PWM were independent of the analysed potentially confounding factors.

### The relationship between masculinity traits and immune functions

The series of multiple regression models showed that, when controlling for these confounding variables that were associated with the dependent variables in the previous analyses (each model had specified controlled factors), the majority of the innate or adaptive immune parameters were not predicted by HGS, SHR, or left or right 2D:4D (Tables 2 and 3). There was only one significant positive relationship between right 2D:4D and T-lymphocyte count (Table 3 and see also S1 Fig in S1 File), but it became non-significant after correcting for multiple comparisons.

**Table 2:** Multiple regression models for innate immunity parameters and masculinity markers.

*Predictors* *Dependent variable*:	max HGS	SHR	Left 2D:4D	Right 2D:4D
*Complement activity*	**β = 0.14; p = .17**	**β = 0.17; p = .12**	**β = -0.10; p = .34**	**β = -0.10; p = .33**
	(R^2^ = 0.01; p < .17)	(R^2^ = 0.02; p < .12)	(R^2^<0.001; p < .34)	(R^2^<0.001; p < .33)
	*f*^*2*^ *= 0*.*021*	*f*^*2*^ *= 0*.*027*	*f*^*2*^ *= 0*.*01*	*f*^*2*^ *= 0*.*01*
*Lysozyme activity*	**β = -0.06; p = .58**	**β = 0.03; p = .77**	**β = -0.01; p = .89**	**β = 0.12; p = .27**
	(R^2^<0.001;p < .58)	(R^2^<0.001;p < .77)	(R^2^<0.001; p < .89)	(R^2^ = 0.002; p < .27)
	*f*^*2*^ *=* 0.004	*f*^*2*^ *= 0*.*001*	*f*^*2*^ *= 0*.*0002*	*f*^*2*^ *= 0*.*013*
*ROS production* ^1^	**β = 0.04; p = .67**	**β = -0.02; p = .85**	**β = 0.04; p = .67**	**β = -0.006; p = .95**
	(R^2^ = 0.12;p < .003)	(R^2^ = 0.12;p < .003)	(R^2^ = 0.12; p < .003)	(R^2^ = 0.12;p < .003)
	*f*^*2*^ *= 0*.*17*	*f*^*2*^ *= 0*.*17*	*f*^*2*^ *= 0*.*17*	*f*^*2*^ *= 0*.*17*
*Phagocytic uptake (N = 81)*	**β = -0.04; p = .73**	**β = 0.06; p = .58**	**β = -0.06; p = .58**	**β = -0.13; p = .24**
	(R^2^<0.001;p < .73)	(R^2^<0.001;p < .58)	(R^2^<0.001; p < .58)	(R^2^ = 0.005; p < .24)
	*f*^*2*^ *= 0*.*002*	*f*^*2*^ *= 0*.*004*	*f*^*2*^ *= 0*.*004*	*f*^*2*^ *= 0*.*017*

Statistics for models (adjusted R2 and p values) are presented in parentheses, effect size (f^2^)

1 controlled for season of study and sports activity

**Table 3:** Multiple regression models for adaptive immunity parameters and masculinity markers.

*Predictors* *Dependent variable*:	max HGS	SHR	Left 2D:4D	Right 2D:4D
*T cell*^*1*^	**β = -0.02; p = .85**	**β = 0.02; p = .81**	**β = 0.05; p = .61**	**β = 0.24; p = .01**
	(R^2^ = 0.15;p < .001)	(R^2^ = 0.15;p < .001)	(R^2^ = 0.16; p < .001)	(R^2^ = 0.21; p < .001)
	*f*^*2*^ *= 0*.*22*	*f*^*2*^ *= 0*.*22*	*f*^*2*^ *= 0*.*23*	*f*^*2*^ *= 0*.*31*
*B cell*^*1*^	**β = 0.008; p = .94**	**β = -0.009; p = .93**	**β = 0.06; p = .53**	**β = 0.10; p = .33**
	(R^2^ = 0.12;p < .002)	(R^2^ = 0.12;p < .002)	(R^2^ = 0.13; p < .002)	(R^2^ = 0.13; p < .002)
	*f*^*2*^ *= 0*.*18*	*f*^*2*^ *= 0*.*18*	*f*^*2*^ *= 0*.*18*	*f*^*2*^ *= 0*.*19*
*IgA levels*	**β = 0.04; p = .69**	**β = -0.13; p = .22**	**β = -0.13; p = .21**	**β = 0.01; p = .92**
	(R^2^<0.001;p < .69)	(R^2^ = 0.006;p < .22)	(R^2^ = 0.007; p < .21)	(R^2^<0.001; p < .92)
	*f*^*2*^ *= 0*.*002*	*f*^*2*^ *= 0*.*017*	*f*^*2*^ *= 0*.*018*	*f*^*2*^ *= 0*.*001*
*IgG levels*^*2*^	**β = -0.05; p = .64**	**β = -0.09; p = .37**	**β = 0.11; p = .26**	**β = 0.01; p = .90**
	(R^2^ = 0.19;p < .001)	(R^2^ = 0.19;p < .001)	(R^2^ = 0.20; p < .001)	(R^2^ = 0.19; p < .001)
	*f*^*2*^ *= 0*.*28*	*f*^*2*^ *= 0*.*28*	*f*^*2*^ *= 0*.*29*	*f*^*2*^ *= 0*.*27*
*Post-vaccination response* ^*3*^	**β = 0.02; p = .85**	**β = 0.11; p = .28**	**β = 0.07; p = .52**	**β = 0.16; p = .12**
	(R^2^ = 0.09; p < .01)	(R^2^ = 0.10;p < .006)	(R^2^ = 0.10; p < .01)	(R^2^ = 0.12; p < .003)
	*f*^*2*^ *= 0*.*14*	*f*^*2*^ *= 0*.*15*	*f*^*2*^ *= 0*.*14*	*f*^*2*^ *= 0*.*17*
*Con A IS (N = 54)*	**β = -0.09; p = .51**	**β = -0.09; p = .52**	**β = -0.03; p = .81**	**β = 0.09; p = .51**
	(R^2^<0.001;p < .51)	(R^2^ = 0.07; p < .52)	(R^2^<0.001;p < .81)	(R^2^<0.001; p < .51)
	*f*^*2*^ *= 0*.*008*	*f*^*2*^ *= 0*.*008*	*f*^*2*^ *= 0*.*001*	*f*^*2*^ *= 0*.*009*
*PWM IS (N = 54)*	**β = -0.01; p = .94**	**β = 0.05; p = .74**	**β = -0.07; p = .59**	**β = 0.001; p = .99**
	(R^2^<0.001;p < .94)	(R^2^<0.001;p < .74)	(R^2^<0.001; p < .59)	(R^2^<0.001; p < .99)
	*f*^*2*^ *= 0*.*001*	*f*^*2*^ *= 0*.*002*	*f*^*2*^ *= 0*.*006*	*f*^*2*^ *= 0*.*0001*

Statistics for models (adjusted R2 and p values) are presented in parentheses, effect size (f^2^)

1 controlled for age, and season of study.

2 controlled for season of study and BMI.

3 controlled for age and fT

When controlling for factors potentially affecting immune parameters such as age, study season, BMI and fT, the results were similar, i.e. HGS, SHR and left 2D:4D did not predict any of the immune functions, whereas T cell was predicted by right 2D:4D (see S2 Table in S1 File).

The relationships between immunity parameters and masculinity markers did not change in the models which were adjusted for body height.

### The relationship between masculinity traits and controlled factors

Because HGS was associated with such controlled factors as a participant's age, BMI and body height (see section "IV" in S1 File, the correlation between body height and BMI was weak (R = -0.22)), we also conducted the analyses adjusted for these. The separate models were carried out for each immune function. These analyses also revealed that HGS is not related to any of the analysed immune parameters.

According to the evolutionary hypothesis concerning the biological implications of sexually selected traits which are commonly perceived as attractive (good genes hypothesis), and are also involved in intra-sexual competition and costly to produce (handicap hypothesis), we assumed that masculinity in men aged 19–36 might serve as a signal reflecting an individual's biological quality. The aim of this study was to check if body masculinity of healthy men is related to immune function, which is one of the most important fitness-related characteristics of an organism, determining risk of morbidity and mortality in all life stages. In general, we found no association between masculinization markers and either innate or adaptive immune responses, even when controlling for a participant's age, BMI, free-testosterone levels, smoking status or sport activity.

The finding in the current study of no associations between SHR or HGS and any of the studied immune parameters, indicates that these indirect markers of pubertal or current testosterone do not reflect immune functioning. To the best of our knowledge, there is no study linking SHR with any of the immune-associated biomarkers, and there have only been limited studies testing the relationship between muscle mass and immunity [45], with only one study directly testing CD4+ count and muscle functions itself as measured by strength [65]. This was, however, only in patients with an HIV-associated immune injury. Raso et al. (2013) [65] showed that disease-associated decreases in HGS are associated with a lower CD4+ count. There are also several studies only indirectly linking HGS with immunity-related characteristics, showing that inflammation-associated diseases [66, 67] or autoimmune disorders [68] are related to lower HGS. Our study is the first to demonstrate that a V-shaped upper body, reflected in SHR and muscle function and measured by HGS, is not associated with immunity in healthy men.

Our results investigating the relationship between masculinity, post-vaccination and lymphocyte-proliferation responses are in contrast to the limited human studies testing ICHH in facial masculinity and the strength of antibody or cytokine response after immune stimulation [e.g. 15, 19]. It is worth underlining, however, that in these two studies the authors analysed only facial (and not body) perception and they did not use anthropometry. Furthermore, Rantala et al. (2012) [15] measured immune response against the hepatitis B virus i.e. conservative antigens, whereas we studied immune response to the influenza virus i.e. a fast evolving/mutating antigen. The lack of association between analyzed markers of masculinization and immune reactivity in response to potentially harmful factors may therefore indicate that the immune response can differ depending on the antigen in question. It is also possible that some vaccine-contained antigens and/or in vitro lymphocyte stimulation may be inadequate to reflect general immune quality. Our results also contradict studies that have indirectly measured immunity (e.g. frequency of infection) and its relationship with facial masculinity (rated and/or morphometric) finding that men with more masculine faces (rated and measured) reported a lower frequency of colds and flu [16, 62]. It is therefore possible that facial dimorphism has more signalling significance than body masculinity (or the traits studied in this paper). On the other hand, Foo et al. (2017a) [63] did not find the relationship between rated facial masculinity and various immune functions measured in saliva. Therefore in the future it will be important for studies to include both facial and body traits related to masculinity in order to address this discrepancy in the literature.

Due to the mixed findings in humans, we should also consider that the immunosuppressive role of androgens (the basis of ICHH) is still controversial. It has been suggested that androgens have immunomodulatory rather than immunosupressive properties [18, 21] and this was also observed in a group of participants included in this study (see S3 Table in S1 File). It may also be the case that in contrast to experimental or in-vitro studies which, in accordance to ICHH, expect a negative relationship between immunity and masculinity, in correlational studies (such as ours) the lack of associations might also be interpreted in the framework of the ICHH and good genes hypothesis. This is because testosterone-induced immune suppression in highly masculine men may suppress immunity to a level similar to that observed in men with a lower immune quality. Consequently, more masculine males might only be a little healthier than average [69], and there may be no noticeable difference in immune quality between those with high and low-masculinity observed in correlational studies. In other words, if, in accordance with ICHH, only individuals with a well-functioning immune response bear a cost associated with testosterone-derived immunosuppression, we should expect that men with a higher expression of masculine traits only have a marginally better, or perhaps a very similar level, of immune quality as men with a lower masculinization level.

Our results are also consistent with recent reports showing that physically attractive traits such as male body height [64] or components of men’s facial attractiveness [63] are not related to immune effectiveness–at least in Western, well-fed societies. The *Immune Priority Hypothesis* (IPH) [64] is another explanation proposed to answer why immune quality might not be reflected by sexually dimorphic traits. IPH suggests that a well-functioning body defence is so crucial for long-lived species, like human beings, who are exposed to many ubiquitous pathogens that immunity cannot be traded for the traits that are not directly related to survival (e.g. body height, SHR or HGS). According to IPH, energy resources should be invested in development of costly morphological signals only when the right amount of energy in creating an optimal immune defence is assured. In this instance, masculine traits instead signal a lack of immune related disorders, and an organism’s ability for sparing additional energy for “luxury” sexual dimorphism. This would mean that despite there being no relationship between immune functions themselves and masculine traits, higher masculinization might still provide information about the biological quality of an organism.

The weak positive association between right 2D:4D (which is a better indicator of prenatal androgenisation than left 2D:4D [70] and lymphocyte T count, might suggest that higher prenatal exposure to androgens is related to lymphocyte count in adults. This result can be partially explained by the organizational action of androgens on the immune system. It was shown that exposure to androgens in early life might permanently affect immunity both in primates [71] and rodents [27], whereas gonadectomy in mature animals does not abolish sex-differences in immune response [72]. This does not mean, however, that testosterone level in adults is not related to immunity. There are several experimental and correlational studies showing that testosterone has a negative impact on lymphocyte T count and/or function [10, 73], and that androgens may increase apoptosis of T cells [74]. It is surprising, however, that our observations concern only the lymphocyte count but not lymphocyte function (the proliferative response). It is also worth noting that our result becomes non-significant after adjusting for multiple comparisons and therefore the obtained relationship between the 2D:4D digit ratio and lymphocyte count should be treated with great caution and further studies are warranted. In general, the lack of associations between masculinity markers and immunity in our study is consistent with the results of a recent meta-analysis that provides no support for immunosuppressive testosterone properties in correlational human studies [18].

There are a few limitations of our study. Since we have studied men from a well-nourished Western population (not a common state in our evolutionary past), one needs to be very cautious with generalizing our results to all ecological conditions humans might have lived in. It is also very likely that a well-nourished urbanized population is not ideal for measuring the masculinity-immune associations due to a relatively low cost associated with immune challenges. Improved living conditions, including hygiene practices, infection prevention (vaccines), and increased access to medications and antimicrobial drugs, have all contributed to the reduction of pathogen exposure and shortening the duration of an infection in these populations. Consequently, the physiological cost associated with the immune system functioning in such a population might be much lower in comparison to a population with a greater pathogen load and a higher risk of infection. Furthermore, the effect size (calculated as a Cohen's f^2^) showed that the magnitude of associations is small or moderate (see Tables 2 and 3), which is also true for a significant relationship between 2D:4D and T cell (f^2^ = 0.31). This indicates that in well-nourished western populations the relationship between immune parameters and masculine traits is relatively low. In other words, the results suggest that the difference in immune functioning between men with more and less masculine traits might be too low to have functional immunological consequences.

The final problem to consider is that of the complicated structure of immunity and the interdependencies between immunity and other physiological aspects. It is likely that the measurements of baseline immune functions in men who declared no health problems, had no chronic diseases or ongoing infections and had a normal level of inflammatory markers (both CRP and WBC) are still inadequate for measuring immune quality. It is possible that, to assess an individual's immune quality, the analysis of many immune parameters, activated in response to real/natural pathogen-inducing infection, should be taken into account. The measurement of an immunological response to pathogen stimulation might be more informative than baseline immune parameters (in a "healthy state", without antigen stimulation) or only vaccine-induced antibody production or mitogen-induced proliferation.

## Supporting information

S1 File Masculinity and immunity. Additional statistical analyses.
(PDF)

S1 Data Masculinity and immunity. Database.
(XLS)
