# Supplementary material for: Masculinity and immune system efficacy in men
Source: PLoS One. 2020 Dec 14;15(12):e0243777. doi: 10.1371/journal.pone.0243777 (PMC7735617; doi:10.1371/journal.pone.0243777)
Supplement: S1 File — Additional statistical analyses. (PDF) [file pone.0243777.s001.pdf]

TITLE: Masculinity and immune-system efficacy in men

Judyta Nowak[1], Barbara Borkowska[1], Bogusław Pawłowski[1*]

SUPPLEMENTARY INFORMATION (SI no 1)

I. Table 1S Inter-correlation between immunity parameters (r Pearsona)

significant correlations were bolded

Immune Parameters	1	2	3	4	5	6	7	8	9	10	11
1. Complement activity	-	0,16	0,04	-0,03	-0,04	-0,06	0,19	0,00	-0,10	-0,10	-0,12
2. Lysozyme activity	0,16	-	0,13	0,07	0,21*	0,22*	0,16	0,19	0,03	-0,04	-0,01
3. ROS production	0,04	0,13	-	0,07	0,09	0,09	-0,03	0,25*	0,12	0,13	0,27
4. Phagocytic uptake (N=81)	-0,03	0,07	0,07	-	0,04	0,01	0,24*	0,08	0,02	-0,06	-0,23
5. T cell	-0,04	0,21*	0,09	0,04	-	0,73**	-0,06	-0,05	0,26*	0,25	0,18
6. B cell	-0,06	0,22*	0,09	0,01	0,73**	-	-0,13	-0,05	0,10	0,13	0,25
7. IgA levels	0,19	0,16	-0,03	0,24*	-0,06	-0,13	-	0,10	-0,06	-0,04	-0,15
8. IgG levels	0,00	0,19	0,25*	0,08	-0,05	-0,05	0,10	-	0,11	-0,40**	-0,39**
9. Post-vaccination response	-0,10	0,03	0,12	0,02	0,26*	0,10	-0,06	0,11	-	0,05	0,15
10. Con A IS (N=54)	-0,10	-0,04	0,13	-0,06	0,25	0,13	-0,04	-0,40**	0,05	-	0,81**
11. PWM IS (N=54)	-0,12	-0,01	0,27	-0,23	0,18	0,25	-0,15	-0,39**	0,15	0,81**	-

* p<0.05 ; ** p<0.01

II. Figure 1S. Relationship between lymphocyte T count and right 2D:4D ratio [N=91]

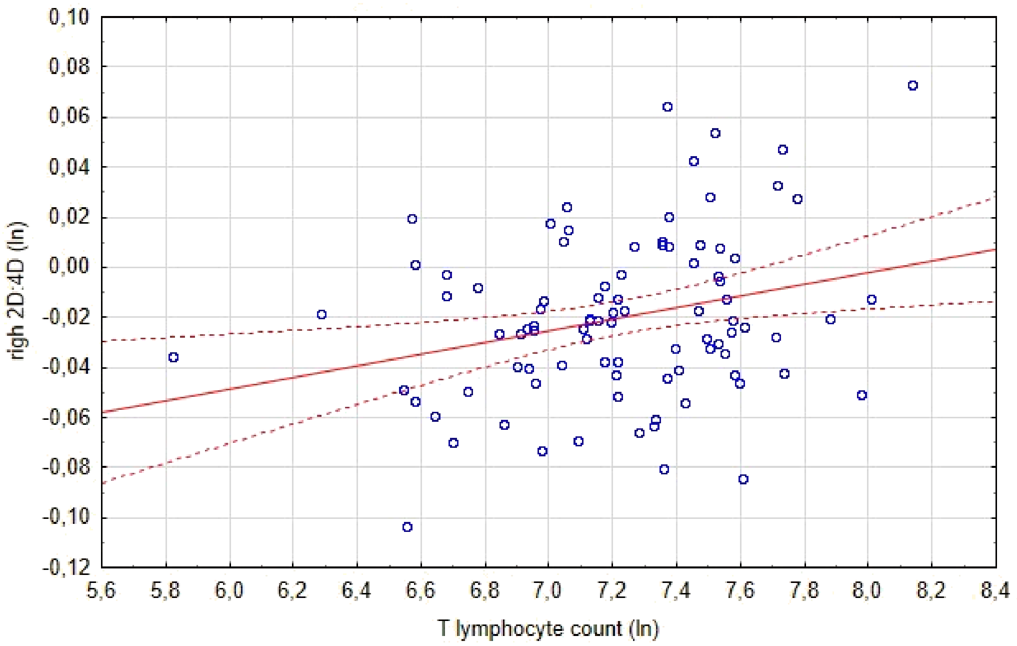

III. Table 2S. Multiple regression models for innate a) and adaptive b) immunity parameters and masculinity markers controlling for age, BMI, study season and fT in each models [N=91]

a)

Predictors / *Dependent variable*	**max HGS**	**SHR**	**Left 2D:4D**	**Right 2D:4D**
Complement activity	**β=0.14; p=.24** (R^2< 0.001; p<.46)	**β=0.18; p=.10** (R^2= 0.01; p<.31)	**β=-0.11; p=.31** (R^2< 0.001; p<.51)	**β=-0.07; p=.54** (R^2< 0.001; p<.61)
Lysozyme activity	**β=0.03; p=.77** (R^2= 0.02; p<.27)	**β=0.04; p=.74** (R^2= 0.02; p<.27)	**β=0.006; p=.95** (R^2= 0.02; p<.28)	**β=0.09; p=.43** (R^2= 0.02; p<.22)
ROS production	**β=0.02; p=.84** (R^2=0.10; p<.01)	**β=-0.05; p=.61** (R^2=0.10; p<.01)	**β=0.006; p=.95** (R^2 = 0.10; p<.01)	**β=-0.001; p=.99** (R^2= 0.10; p<.01)
Phagocytic uptake (N=81)	**β=-0.005; p=.97** (R^2= 0.003; p<.39)	**β=0.03; p=.77** (R^2= 0.004; p<.38)	**β=-0.08; p=.48** (R^2= 0.01; p<.34)	**β=-0.15; p=.18** (R^2= 0.03; p<.22)

b)

Predictors / *Dependent variable*	**max HGS**	**SHR**	**Left 2D:4D**	**Right 2D:4D**
T cell	**β=-0.03; p=.75** (R^2=0.16; p<.001)	**β=0.01; p=.90** (R^2=0.16; p<.001)	**β=0.08; p=.42** (R^2=0.; p<.)	**β=0.27; p=.005** (R^2=0.23; p<.001)
B cell	**β=-0.02; p=.83** (R^2=0.19; p<.001)	**β=-0.04; p=.66** (R^2=0.19; p<.001)	**β=0.12; p=.24** (R^2=0.20; p<.001)	**β=0.14; p=.14** (R^2=0.21; p<.001)
IgA levels	**β=-0.03; p=.82** (R^2=0.004; p<.38)	**β=-0.14; p=.19** (R^2=0.02; p<.22)	**β=-0.17; p=.11** (R^2=0.03; p<.16)	**β=0.04; p=.71** (R^2=0.005; p<.37)
IgG levels	**β=-0.04; p=.72** (R^2=0.21; p<.001)	**β=-0.10; p=.32** (R^2=0.22; p<.001)	**β=0.08; p=.41** (R^2=0.22; p<.001)	**β=-0.01; p=.91** (R^2=0.21; p<.001)
Post-vaccination response	**β=0.03; p=.77** (R^2=0.09; p<.02)	**β=0.12; p=.26** (R^2=0.11; p<.01)	**β=0.07; p=.51** (R^2=0.10; p<.02)	**β=0.13; p=.21** (R^2=0.11; p<.01)
*Con A IS (N=54)***	**β=-0.09; p=.53** (R^2<0.001; p<.61)	**β=-0.14; p=.34** (R^2<0.001; p<.52)	**β=-0.004; p=.97** (R^2<0.001; p<.68)	**β=0.15; p=.30** (R^2<0.001; p<.50)
*PWM IS (N=54)***	**β=0.004; p=.98** (R^2<0.001; p<.70)	**β=0.02; p=.91** (R^2<0.001; p<.69)	**β=-0.04; p=.75** (R^2<0.001; p<.68)	**β=0.05; p=.73** (R^2<0.001; p<.67)

* controlling for age, BMI and fT

IV. The relationship between masculinity-related traits and controlled factors

HGS: Week significant associations between HGS and both participant's age (β=0.39; p<0.01), BMI (β=0.26; p=0.01) and body height (β=0.38, p<0.01) were observed, whereas free testosterone (β=-0.09; p=0.39), smoking status (β=-0.02; p=0.82) and sports activity (β=0.06; p=0.54) were not related to HGS.

SHR was not related to any of the controlled factors: participant's age (β=0.04; p=0.69), BMI (β=0.17; p=0.10), free testosterone (β=0.03; p=0.79), body height (β=-0.04; p=0.71), smoking status (β=0.17; p=0.10) or sports activity (β=-0.04; p=0.71).

Right 2D:4D was not associated with any of the controlled factors: participant's age (β=0.06; p=0.58), free testosterone (β=0.09; p=0.39), BMI (β=-0.08; p=0.45), body height (β=0.11; p=0.29), smoking status (β=0.13; p=0.22) or sports activity (β=0.12; p=0.24)

There was also no relationship between **Left 2D:4D** and participant's age (β=0.13; p=0.21), free testosterone (β=0.14; p=0.19), BMI (β=0.005; p=0.97), body height (β=0.12; p=0.26), smoking status (β=0.14; p=0.18) or sports activity (β=-0.01; p=0.94).

V. Inter-correlation between HGS, SHR, right 2D:4D and left 2D:4D

Only weak correlation was observed between HGS and SHR (r=0.22, p=.04) as well as right 2D:4D and SHR (r=-0.21, p=.05), whereas right 2D:4D was moderately correlated with left 2D:4D (r=0.53, p<.001).

VI. Table 3S. The correlation between immune parameters and fT

Immune Parameters	free testosterone (r Pearsona)
1. Complement activity	-0,034
2. Lysozyme activity	0,056
3. ROS production	0,158
4. Phagocytic uptake (N=81)	0,111
5. T cell	-0,089
6. B cell	-0,188
7. IgA levels	0,055
8. IgG levels	0,203
9. Post-vaccination response	**0,242***
*10. Con A IS (N=54)***	0,035
*11. PWM IS (N=54)***	0,010

*p<0.05
